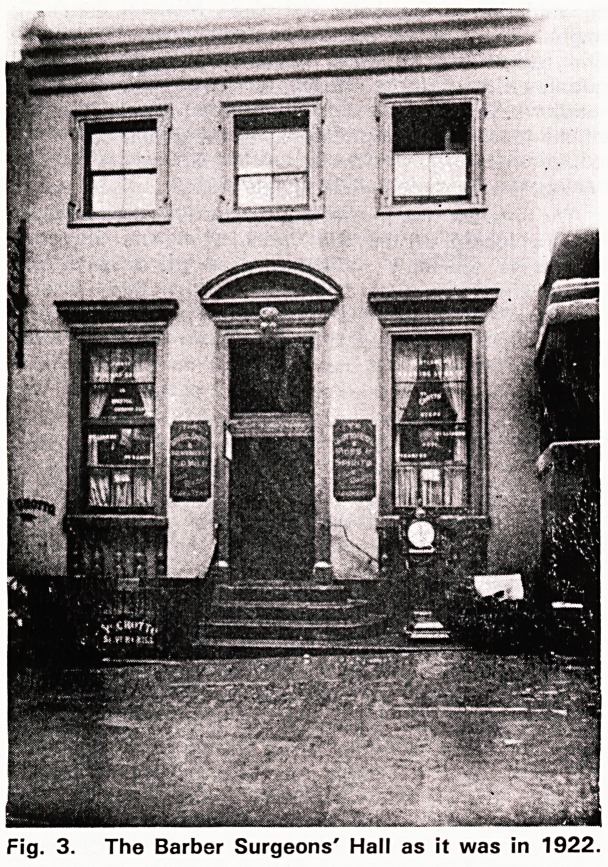# The Barber Surgeons of Bristol

**Published:** 1975

**Authors:** R. Milnes Walker

**Affiliations:** Professor Emeritus of Surgery, University of Bristol


### The Barber Surgeons of Bristol

Part of a lecture given on 18th September 1975, on the occasion of the 75th anniversary of the founding of Southmead Hospital.

**R. Milnes Walker**
Professor Emeritus of Surgery, University of Bristol

The association of surgeons with barbers goes back to the early middle ages. At the time of the Norman Conquest when the Church was the dominating factor in western Europe and practically all educated people were members of the clerical profession, writing and knowledge were the province of ecclesiastics either in association with the monasteries which were springing up particularly in France and England, or with the cathedrals. At that time the priests were with few exceptions the only people who could read Latin and this precluded others from access to such medical texts as were available. Salerno was the only secular medical centre, but Montpelier was to follow in the next century. Thus any medical treatment which was available was performed by the priests, especially those who were in the monasteries. In the twelth century the authorities of the Church became alarmed at the increasing medical activities of the priests, particularly those in higher orders where any practice of surgery might lead to the shedding of blood, and a priest who had blood on his hands was officially debarred from the higher offices of the clerical heirarchy.

This state of affairs led to a series of enactments. The Council of Rheims in 1134 prevented the presence of priests and monks at public lectures on medicine. At the Council of Tours in 1163 Pope Alexander III decreed that clergy were not to undertake any surgery, and finally in 1215 the Fourth Lateran Council disallowed all priests, deacons or subdeacons from the practice of surgery under penalty of excommunication, and a few years later Pope Honorius III prohibited them from the practice of medicine. Thus it came about that treatment was only carried out in the monasteries by those in lower orders, and as the barbers were accustomed to the use of the razor, they were the people who carried out any surgical treatment, perhaps under the direction of the priests. As time went on the knowledge of surgery which was then available spread from the monasteries to the barbers outside. It was particularly in time of war that there was a demand for the services of those who had experience in the treatment of wounds, thus the barbers were called upon to accompany the armies. Their services were also required at tournaments where often severe wounds were inflicted, and this introduced them to the more aristocratic members of the community who took part in such events. In fact the first mention of a King's surgeon in England is in 1235 when Master William, the King's Surgeon, was presented to the living of a church in the Diocese of Lincoln, thus indicating that he was in holy orders, and the edicts of the Church of Rome were not being strictly enforced in the country.

It was during the thirteenth century that trade gilds first became a feature of cities in England, and Bristol, with its population at that time only second to London, was early in the field. The first reference to the Company or Gild of Barber Surgeons in London is in 1308, but we have no knowledge of such a gild in Bristol before 1395, though in both cases the Gild had probably been in existence for some time, but earlier manuscripts relating to them have not survived. The gilds were originally concerned with the standard of work of their members, but at the same time they established a closed shop which they rigorously attempted to retain as we shall see when I deal with their later history.

Most of these gilds sought recognition by the municipal authorities to whom they could appeal to try and enforce their regulations, and particularly their monopoly. In 1370 an Act of Parliament decreed that there should be an apprenticeship for every craft for seven years. It thus became the province of the craft gilds to see that such apprenticeship was carried out, and that those who had served their apprenticeship were of the required standard. The first attempt to regulate the practice of medicine in this country was in 1421, when Parliament decreed that only those who were licensed by a University could practise medicine, and surgeons had to be licensed by Masters of the Art,

**Fig. 1. The earliest reference to the Barber Surgeons' Company in the Little Red Book, 1395.**

and this in effect meant the Masters of the Gilds. In 1436 the Ordinances of all gilds had to be submitted to the magistrates of each town for ratification with the result that Barber Surgeons' gilds in London, York, Bristol, Newcastle, Salisbury, Exeter and Durham applied for municipal licences. It is reported that there were gilds of Barber Surgeons in at least twenty-five cities in the British Isles. The licence was granted to the Bristol Company in 1439.

I have already mentioned the first reference to the Bristol Gild of Barber Surgeons in 1395, and this is to be found in the Little Red Book which is in the City Archives (Fig. 1). It gives an insight to the methods and customs of the gild so I quote it in full. It is written in Latin, but I give the translation by Mr. F. B. Bickley.

"Ordinance for the Barbers in the time of William Frome, Mayor. On Wednesday on the morrow (of the feast) of the Exaltation of the Holy Cross (14th Sept.) in the 19th year of the reign of King Richard II after the conquest (1395) it is ordained both by William Frome, Mayor of the town of Bristol, and John Stephens then Sheriff of the town, and by the forty men who have rule of the town by virtue of the charter of liberty of Lord Edward late King of England to the aforesaid town, and also of the consent of John Chiltenham, John Colchestre, John Laurence, John Stephenes, Barber, John Barbour, Adam Bony, John Kent, John Wolf, John Pycot, Henry Faryndon, Richard Barbour and John Wymbourne, Barbers, assembled for the government of their craft aforesaid, and by their unanimous assent, it is agreed that no one of the aforesaid craft of whatever state or grade he shall be shall exercise within the liberty of the town aforesaid his craft in tonsure nor in anything pertaining to the same craft on Sundays in any way, strangers coming suddenly to the said town, and the six Sundays in autumn and the Sunday next before the Nativity of our Lord when it shall happen that the day of the Nativity of our Lord is on Monday the morrow of that Sunday, only excepted. Provided however that the money coming from the aforesaid strangers on the aforesaid Sundays, and six pence for the aforesaid six Sundays in autumn be distributed among the needy and poor by the masters of the craft aforesaid for the time being. And so often as any of the aforesaid craft shall have failed in aught or acted contrary to the aforesaid ordinances he is bound to pay to the Community of the town of Bristol by distraint and judgement of the Mayor of the town aforesaid for the time being by ways and means which can be better known and employed 20d., and as much to the masters of the craft aforesaid for the time being by distraint and judgement of the Mayor aforesaid for the time being as is aforesaid."

The wording of the ordinance suggests that the gild had been established well before 1395, and indicates that it was governed by a Court of twelve men, all barbers. Presumably the special note of "barber" after John Stephenes' name is to distinguish him from the sheriff who had the same name. The fact that two of them had the surname of Barbour is, of course, an indication of the common practice of naming people by their trade, this being the origin of many family names. It will be noticed that the Company here is always that of barbers and there is no mention of surgeons. In fact all the records which survive make no mention of surgeons before 1561, as I shall relate later.

The question of practising barbery on Sundays was one which caused frequent trouble, and even as late as 1767, when the Company was no longer very active, there is a record that in consequence of frequent prosecutions of barbers for shaving on Sundays the Company gave notice that their shops would not open on Sundays and that recalcitrant journeymen who were not members of the Company would be warned that the parish constables would take note if they failed to attend divine services.

The next new ordinances were introduced in 1418 and are described in the Little Red Book as "Ordinances of the Craft of Barbers made anew" when Robert Fussell was Mayor and John Colchestre was Master of the Gild. They are written in French. The main complaint which led to these ordinances was that "unlearned people" such as tailors, weavers, fullers, mariners, smiths, cordwainers and others were infringing the rights of the barbers by polling and shaving and the barbers were losing their profits. These new ordinances also laid down that no apprentices were to be taken for less than seven years thus complying with the Act of Parliament of 1370. No member of the craft was to take a servant "learned and cunning in the same craft" for less than a year unless the servant was a stranger, that is to say, not a citizen of Bristol, who was passing through the town, and he could be taken on by the day or week. No member was to purloin servants from other members. No one was to keep a barber's shop unless he was a freeman of Bristol, and no one was to ply his craft except openly in a shop. Fines were laid down for any infringement of the ordinances and these were to be divided equally between the Gild and the Common Council. The Council approved these ordinances, thus giving the members of the Company a monopoly and the Guild now had full legal status.

The next reference which we possess is again in the Little Red Book and is dated 1439. It contains the full ordinances of the Gild, occupying six pages, and this time they are written in English. Reference is made to the two previous groups of ordinances which had already been approved by the Mayor and Council, and new ones were added as follows.

The Gild should elect at the time of the feast of Michaelmas, a Master and two Wardens, who will be sworn in by the Mayor, Sheriffs and Bailiffs. The members of the craft were to take part in the general procession at the feast of Corpus Christi as had been the custom before that time, and if, having been warned by the Master and Wardens, they did not take part, they were to be fined 12d. to the City Fund and 4d. to the craft. Members had to pay 8d. to the Common Fund and 4d. to the craft if they took on any servant, 12d. (6d. to each) for an apprentice whose indentures had to be sealed, the fee for this being 6s. 8d., 40d. each to the Common Fund and 40d. to the craft. Strangers coming in as apprentices had to pay 2s. similarly divided. It was ordered that the ordinances should be enrolled in the red paper of the Gildhall among the ordinances of other crafts. The Mayor and Common Council retained the right to revoke the ordinances at any time. The common seal of the town was appended to the ordinances on the Tuesday next after the feast of the Apostles Philip and James (May 1st) in the 17th year of the reign of King Henry VI after the conquest (1439).

The next reference is found in the Great Red Book and is an interesting one. It states that on the feast of St. John and St. Peter in 1430, when William Cannynge was Mayor the Barbers and the Wax makers were allocated 4 gallons of wine. It has been suggested that these two crafts were united as was the case in some of the other provincial cities, e.g. Newcastle, Chester and Norwich. There is no mention of the Waxmakers in the Little Red Book, and if there was any collaboration between the two crafts is must have ceased before the middle of the next century.

We now have a gap of a century before there is any surviving document relating to the Gild. In August 1532 Thomas Rogers, described as a barber surgeon, received the Freedom of the city, this being the first mention of a surgeon.

**Fig. 2. The Lease of the Barber Surgeons' Hall to the Company in 1561.**

The next surviving document gives the first reference to a hall. It is the indenture of a lease of a hall to the company, dated 1561, and is in the City archives (Fig. 2). It begins:—"This Indenture made the 13th day of August in the third year of the reign of our Sovereign Lady Elizabeth by the Grace of God of England, France and Ireland Queen Defender of the Faith between John Cuff of the City of Bristol merchant on the one part and John Thompson of the same City barber now being master of the craft or mystery of barber surgeons and physicians of the said City of Bristol." It then goes on to describe the property to be leased as "All that hall called and known by the present name of the barbers hall situate lying and being within the city of Bristol foresaid in a certain place called Court Place in a lane leading from Corn Street to Saint Nicholas Street situate in and between the ground of Robert Saxon alderman on the one part and the grounds of the said John Cuff on all other parts and doth contain in length 27 feet and in breadth 20 feet, together with the chamber over the said hall and the same is now at this present standing and being in an ample and large a manner as the said now hold". The lease was for three score years. The rent was to be 16s. paid in equal instalments at the feast of the annunciation of Our Lady and of St. Michael the Archangel. The landlord was to be responsible for repairs and had to see that the premises were dry and the gutters without leaks. If the rent was in arrears the landlord could distrain on the premises, but in the case of the lease being terminated, the master and wardens could remove their property, but had to leave a window facing Mr. Saxon's sufficiently glazed.

The interest lies in that this is the first time that we have a record of the Gild being of Barber Surgeons and it is the only indication that there were also physicians who were members of the Gild. It also suggests that the Gild already had the hall on a previous lease as it was already known as the Barber Surgeons Hall. It was near, but not on the same site as the one which we know the Gild occupied later on.

In the papers of the municipal charities we find that the building was leased in 1620 to the Barber Surgeons by John Whitson, a prominent merchant in Bristol, being described as in Court Place in a lane out of Corn Street. The lease was for 41 years at a rent of 20s., a stipulation being made that the premises were not to be used for a school, for victualling or as a tippling house. In 1656 the lease was renewed until 1697 at a rent of 40s. The size of the site is given as 27 by 25 feet and mention is made of an upper chamber so this is almost certainly the same property as that mentioned in the lease of 1561. A deed of 1699 records that Messrs Jones and Burgess are to have the room formerly called the Barbers' Hall, and the room over it situate over the cellars of Mr. T. Burgess that go athwart Cypher Lane which was the lane mentioned in the 1561 lease but not by name. This leads us to think that before 1699 the Company had a Hall of their own. It is known that later they had a Hall in Exchange Avenue, which they sold in 1790, and which was destroyed by enemy action in 1940. The architecture of this building (Fig. 3) would fit in with its construction at the end of the seventeenth century, so it was probably built about 1697 when the lease of the premises in Cypher Lane expired, and was built specially for the Company. It is shown on Rogues' plan of Bristol in 1742 as the Surgeons Hall. The site of this Hall measured 40 by 25 feet, and had frontages both on Exchange Avenue and on Shannon Court, so it was considerably larger than the one which the Company had previously leased.

The use of this new Hall by the Company was short lived, as by the middle of the eighteenth century the Company was beginning to disintegrate. In 1745 Andrew Hooke took a lease of the premises. He was evidently a colourful character who had been in prison for debt. In 1743 his wife opened a Coffee House, called St. Michael's Coffee House in Maudlin Street. For a time Hooke was a journalist who, from 1742 to 1749 published a journal called "The Oracle", and he gave lessons in geography. When he took the lease of the Barber Surgeons Hall he opened it as a coffee house, at first Hooke's Coffee House, but later calling it the West Indian Coffee House. In 1750 the property was mortgaged by Isaac Page (Master) and other members of the Company to Standfast Smith, an apothecary and Charity Smith, spinster, for £500. In 1759, when Thomas Ellis was Master, the Company bought the ground rent of £4 on part of the property. In the City archives is an indenture dated 1755, made

**Fig. 3. The Barber Surgeons' Hall as it was in 1922.**

between Samuel Pye, Thomas Hollier, Samuel Tipton and Isaac Page, described as "surviving feoffees of all and singular the Lands Tenements and Hereditaments belonging or reputed to belong to the Society or Company of Barber Surgeons". The property is described as "Surgeons Hall, Committee Room and Anatomical Theatre" which again suggests that the building had been erected for this purpose. At that time the West Indian Coffee House was occupied by Elizabeth Young, widow. The members of the Company are described as members who have served the office of Master. Thomas Farr was given possession for one year.

In 1767 when T. Parsley was Master, he and the other feoffees arranged another mortgage for £100 which was advanced by Standfast Smith. In 1775 the property was assigned to a W. Baker of Tickenham by the Master, Charles Armitage and the other feoffees, and finally it was sold to Matthew Wright, a merchant, in 1790. When Dr. Parker wrote in 1922 it was a licensed house called "The Grapes".

But now it is necessary to go back to the sixteenth century to fill in the gaps. In 1599 new ordinances, which for the first time applied particularly to surgeons, were submitted to the Common Council, but they were not confirmed until January 1611-12. They were as follows. No one was to practice surgery before he had been to the Surgeons Hall and passed as skilled enough unless it is in an emergency or something done free for a poor body. This is the first mention of surgeons being examined at the Hall though undoubtedly it had been the practice since the Parliamentary decree of 1421.

No surgeon was to take a cure out of another's hand unless called in by two "indifferent persons", one chosen by the surgeon the other by the patient.

Anyone attempting a cure should never neglect or give up a case.

In the case of a poor man needing a cure the Master of the Gild was to detail a surgeon who on completion of the cure was to present an account to the Master who would decide how much the patient could pay. The surgeon was to get nothing if the patient was unable to pay.

Further new ordinances were found necessary in 1650-51 which dealt with ships' surgeons, and no doubt were associated with the increasing importance of Bristol as a port. They are concerned with the equipment required.

Every sea surgeon was to give notice to the Master of the Company in order that he may view his chest before it goes aboard. There was a fee for viewing, 2/6d. for city freemen and 3/4d. for strangers. If a surgeon refused to have his chest inspected he was to be fined 6/8d., if a freeman, otherwise 10s. The Master had to view every chest within 24 hours of receipt of notice from the surgeon. Every chest once passed was to be locked and the key given to the ship's master and if he did not approve of the contents the final decision was to rest with the Mayor and Aldermen.

Six years later it was ordered that no apprentice could sign on as a surgeon's mate unless he had served three years of his apprenticeship and had been approved by the Master and Wardens of the Gild.

Dr. Parker and Mr. Pountney looked through the Burgess books of the City of Bristol from about 1545 to 1745, and found that during those two centuries there were 500 admissions to the Gild, but there is no note as to whether they were barbers or surgeons. It thus seems that there was an average of two or three admissions to the Company each year. During the same period 250 apothecaries and 36 physicians are listed.

In 1652 a completely new set of ordinances were submitted to the Mayor, Aldermen and Common Council of the City, and these have been reproduced in full by Dr. Parker. Of the 23 clauses many include the fees for services or the fines for not obeying the ordinances. There was to be a meeting each year on September 16th for the election of the Master and Wardens, but if this fell on a Sunday the meeting was to be held on the following day. Any member who failed to attend without reasonable excuse was to be fined ten shillings. For not attending quarterly meetings or paying quarterage the fine was 3/4d. As was a common rule in gilds if the Master or Wardens declined to take office when elected, they were fined, and in this case the fine was 40s. for the Master and 20s. for the Wardens. Fines in all these cases were to be divided equally, half to the Mayor for the use of the poor and half to the Company.

Ordinance five deals with apprentices who were to be taken for seven years, and their indentures entered at the Tolsey of the City. Masters and past masters could take three apprentices at a time, other members only two. The fine for not entering the indentures was 40s., for taking more than the allowed number 20s.

plus 3/4d. a day. Journeymen strangers, that is persons who had completed their apprenticeship but had not been elected to the Company and were not citizens of Bristol could only be employed for one year, no more, no less, and permission had to be obtained from the Mayor. Journeymen who were citizens could be employed by leave of the Master and Wardens. If permission was not obtained the fine was 40s. for every month of employment. Journeymen paid 2/6d. on taking up employment; on taking an apprentice the member of the Company paid 12d.

The monopoly was maintained, no one being allowed to open a barber's shop unless he was a member of the Company and a burgess of the City, and the fine for infringing this right was 20s. for each week. No one was to be admitted to the Company unless he had served his seven years of apprenticeship, unless the whole Company should agree. If this rule was broken the Master had to pay the large fine of £20. There was the usual rule about Sunday trading, the fine being 6/8d. for each offence.

The next four clauses deal with surgery and follow the ordinances of 1599. No member was to take in hand any cure unless he had shown himself as being sufficiently skilled to the Master, but what sort of test was to be made is not related. The fine for infringement was 40s. For accepting the patient of another member without the proper procedure the fine was 20s. The next clause sets an ethical standard—"No person of the Company taking in hand any cure shall suffer such a cure to perish for want of looking into, but shall do his uttermost endeavour and give due attendance upon every such patient as he shall take to hand to the best of his power and skill until such a cure be ended, upon pain to forfeit for every such offence £5." Members were not to use any "approbrious unseemly or uncomely words to anyone that is or has been or shall be Master of the Company, and to be further dealt with as the Mayor of the City shall think fit." There is an ordinance relating to consultations in serious cases—"If a patient is in danger of life or limb, a member who has undertaken a cure must inform two or three of the chiefest and most skillful therewith, who may view the same cure before he take it in hand." The fine for failing to do this was £5. The ordinances relating to ship's surgeons which had only been introduced in the previous year were retained, but sea chests had to be examined by the Master and two other members of the Company.

It will be noted that some of these ordinances apply equally to all members of the Company, while others concern only barbers, others only surgeons. This distinction between the two different groups in the Company had gradually crept in, but there is no indication in the Bristol Company's records that this was officially recognised. In London the position was quite different. Here the Act of Parliament of 1540 recognised this distinction for it laid down that there were to be four wardens, of whom two were barbers and two were surgeons, so that in effect the Master was a barber or a surgeon in alternate years, and this continued until the surgeons left the Company and formed their own Company in 1745.

The ordinances of 1652 were approved by the Mayor, Aldermen and Common Council on 17th April, and they gave permission for the Master to bring an action in the Court of Record held before the Mayor and Aldermen for the recovery of any of the fees or fines.

In 1663 the Gild complained to the Common Council that apprentices were being taken on illegally. Seven years later there was another petition to the Council for protection from the Chancellor of the Diocese who had invoked a statute of 1511 on the grounds that the Bristol Barber Surgeons were without licences issued by his office and therefore were not qualified to practice surgery. The Chancellor, Henry Jones, threatened to take action against them but in September of that year the Council, who were not on very good terms with the ecclesiastical authorities, decided to defend the surgeons and nothing more was heard of the matter.

In 1673 a further protest was made to the Mayor that some members were engaging more than the number of apprentices allowed by regulation.

The next reference which we have relates to the founding of the Royal Infirmary when there was evidently the beginning of the differences between the surgeons and the barbers. The first moves to found the Infirmary were made in 1736, when, in November of that year, a subscription was opened for the purpose of the erection of the building. The first meeting of the Board of Trustees was held in the Council House on 7th January 1736-7, and a Committee was set up which met weekly. Its first meeting was on January 14th and Monro Smith in his history of the Infirmary gives the names of those who attended. The meetings were held either at Forster's Coffee House which was next to the Council House in Corn Street, or at Mrs. Barry's Coffee House or at the Surgeons' Hall, which, as we know, was only a few yards away.

On 4th February 1736-7 at a meeting of the Committee at the Surgeons' Hall, John Eldridge was elected Treasurer, and at another meeting in the Hall on 20th May in the same year the first two surgeons, William Thornhill and Thomas Page were appointed, receiving 36 and 30 votes respectively. At another meeting in the Hall in the following October the first matron was appointed at a salary of £15 a year.

The surgeons in Bristol at that time had learned their profession by apprenticeship to barber surgeons. One such member was J. Rosewell, more usually known as Old Rosewell. He had a large practice at a shop in All Saints' Lane. At the door he displayed a staff, a porringer and a red garter, the insignia of his trade, and here his apprentices learned to shave, to bleed and to draw teeth. He did not recognise the rule about Sunday trading for it is related that outside his shop on Sunday mornings were swarms of people to be bled for which they paid between six pence and one shilling. In 1750 he was one of the members of the Company who mortgaged the property to Mr. Standfast Smith, and he died ten years after this.

William Thornton, the first surgeon to be appointed to the staff of the Infirmary had been an apprentice to a W. Shepherd, and was admitted to the Freedom of the Company in 1719. An apprentice of Rosewell's was William Barrett who applied unsuccessfully for a vacancy on the staff of the Infirmary in 1759 caused by the resignation of James Ford. Ford had been admitted to the Company in 1739 and was elected to the Infirmary staff in 1743, having also been an apprentice of Rosewell.

The other surgeon appointed to the staff in 1737, Thomas Page, had been admitted to the Freedom of the Company in 1716. He died in 1741 and his place was taken by his son, John, who had been admitted to the Company in 1737. He and James Ford are known to have given lectures on anatomy at the Surgeons' Hall in 1744, 1746 and 1747, in spite of the fact that, as has been mentioned, the Hall was leased to Hooke in 1745. John Page styled himself in 1741 as Praelector in Anatomy, but there is no indication that this was an official title.

Two of the members of the Company, Thomas Hollier and Samuel Pye, who signed the indenture letting the Hall to Thomas Farr in 1755, and who are described as having served the office of Master, were both surgeons to St. Peter's Hospital, the former from 1743 to 1753, and the latter from 1713 to 1736. The relationship of Isaac Page, a barber who signed the indenture, to Thomas and John Page is not clear.

The fees charged by Rosewell for bleeding have already been mentioned, but competition for trade must have been keen for in Felix Farley's Journal of 9th March 1754 was an advertisement as follows:— "Henry Harnes, barber, Redcliff Pit, shaves each person for two pence, cuts hair for three half pence and bleeds for six pence. All customers who are bled he treats with two quarts of good ale, and those whom he shaves or cuts their hair with a pint for each."

Shaving on Sunday still seems to have been a bone of contention. In the same journal of 18th November 1758 there is a note "Whereas at the swearing in of the Master of the Company of Barber Surgeons the Rt. Worshipful, the Mayor, was pleased to take notice to them of the scandalous practice of shaving on the Lord's Day desiring the same to be suppressed." This indicates that as late as 1758 the Masters of the Company were sworn in by the Lord Mayor, though 1743 was the last time that the Company appeared in a civic procession, marching, so it is related, with music, on the occasion of the opening of the Exchange on September 21st.

The first definite indication of the disruption of the Company came in 1739 when Isaac Page and 31 other peruke makers and barbers, freemen of the Barbers Company, presented a petition to the City Council complaining of divers impositions and grievances inflicted by their surgical brethren. A counter petition was produced by the Master and Wardens of the Company expressing surprise that some uneasie members should importune the Chamber with such ill-founded discontents. The documents were referred to a committee, and as still happens when such a course is followed nothing more was heard of the matter.

It is noted that by 1793 there were in Bristol 20 surgeons and 35 apothecaries.

In London the Surgeons left the Barber Surgeons Company in 1745, this being done by Act of Parliament. The surgeons then formed their own Company which was replaced by the Royal College of Surgeons in 1800. Things were quite different in Bristol, where about the time of the establishment of the Infirmary the surgeons seem gradually to have left the Company, particularly as it was no longer possible to enforce the strict monopoly which the Company had in previous times. So the Company seems to have gradually faded out, and what happened to its possessions, other than the Hall it has been impossible to trace.

When Dr. Parker wrote on the subject he traced the only known moveable possession of the Company and this is dated 1769. It is a silver tobacco box, inscribed "The Gift of Mr. Standfast Smith, 1769" and "Thomas Ellis, third time Master". It carries, beautifully engraved, the Arms of the Barbers Company of London, which had been granted to them in 1569. The provincial Barber Surgeons Companies do not appear to have had arms of their own, but the London ones were used by the Companies of Dublin, Newcastle and Chester, and in this example of Bristol. In 1922 the box was in the possesion of Mr. W. D. Fripp.

Standfast Smith was an apothecary, practising in Corn Street, but he was also apothecary to St. Peter's Hospital, and had taken the mortgage together with Charity Smith in 1750 of the Hall when Isaac Page was Master. The others who signed on behalf of the Company were J. Deverill, S. Pyke, W. Hargest, J. Rosewell sen., J. Hillier, Alex. Morgan and Sam. Tipton. Thomas Ellis was admitted to the Company in 1739, and was Master in 1750, 1759 and 1769.

As has been mentioned, the last time that we hear of the Company is the sale of the Hall to Matthew Wright in 1790. The deed relates that the only remaining members of the Company were Charles Armitage, Master and peruke maker, T. Parsley, Barber Surgeon and Master in 1757, John and Philip Crocker, and two other peruke makers, only six remaining of what must have been in earlier times a flourishing Company. The Hall was sold for £900, and whether this was divided amongst the few remaining members or was required to pay debts, we do not know. Standfast Smith was now dead, but presumably the mortgage of £600 had to be paid off to his executors. Thus came to an end a Company which had been in existence for at least 400 years, but which had ceased to have a practical place in the changing life of the City of Bristol.

::————————::

The main sources of references are:—

The Little Red Book of Bristol, ed. F. B. Bickley, 1900.

The Great Red Book of Bristol, Bristol Record Society, vol. 4. 1930.

R. Theodore Beck. The Cutting Edge. 1974.

J. Latimer. Annals of Bristol. 1900.

George Parker. Transactions of the Bristol and Gloucestershire Archaeological Society, vol. 4. 1922.

F. H. Rogers. The Bristol Craft Guilds. Thesis, Bristol University. 1949.

G. Monro Smith. History of the Bristol Royal Infirmary. 1917.

::————————::

I wish to thank particularly Miss Mary Williams, City Archivist, for access to the Archives, and permission to photograph some of them. Also thanks are due to Mr. John Barker for transcribing the 1561 lease of the Barber Surgeons' Hall, and to Mr. J. A. Eatough of the University Department of Medical Illustration for the photography.

## Figures and Tables

**Fig. 1. f1:**
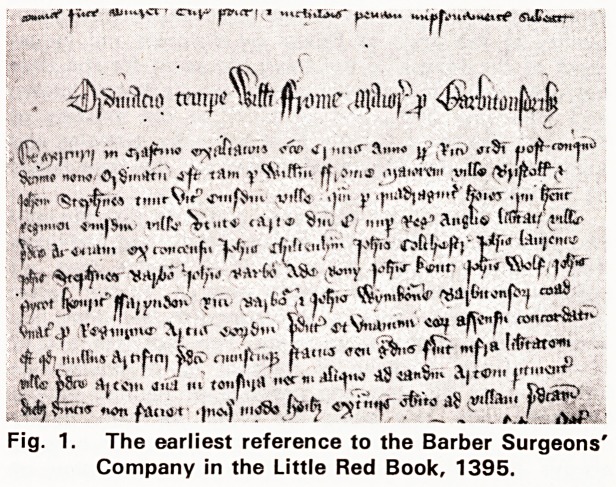


**Fig. 2. f2:**
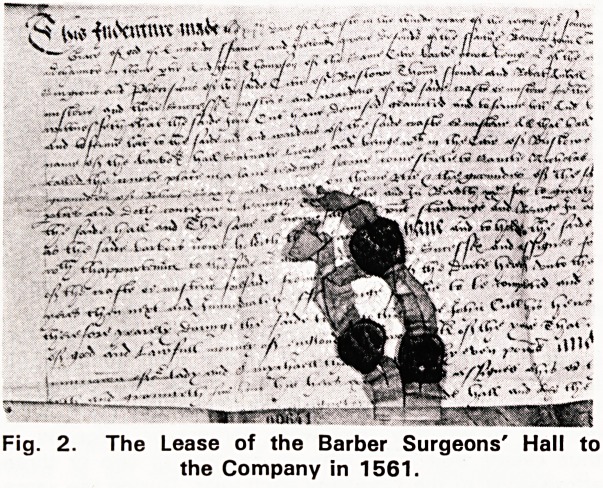


**Fig. 3. f3:**